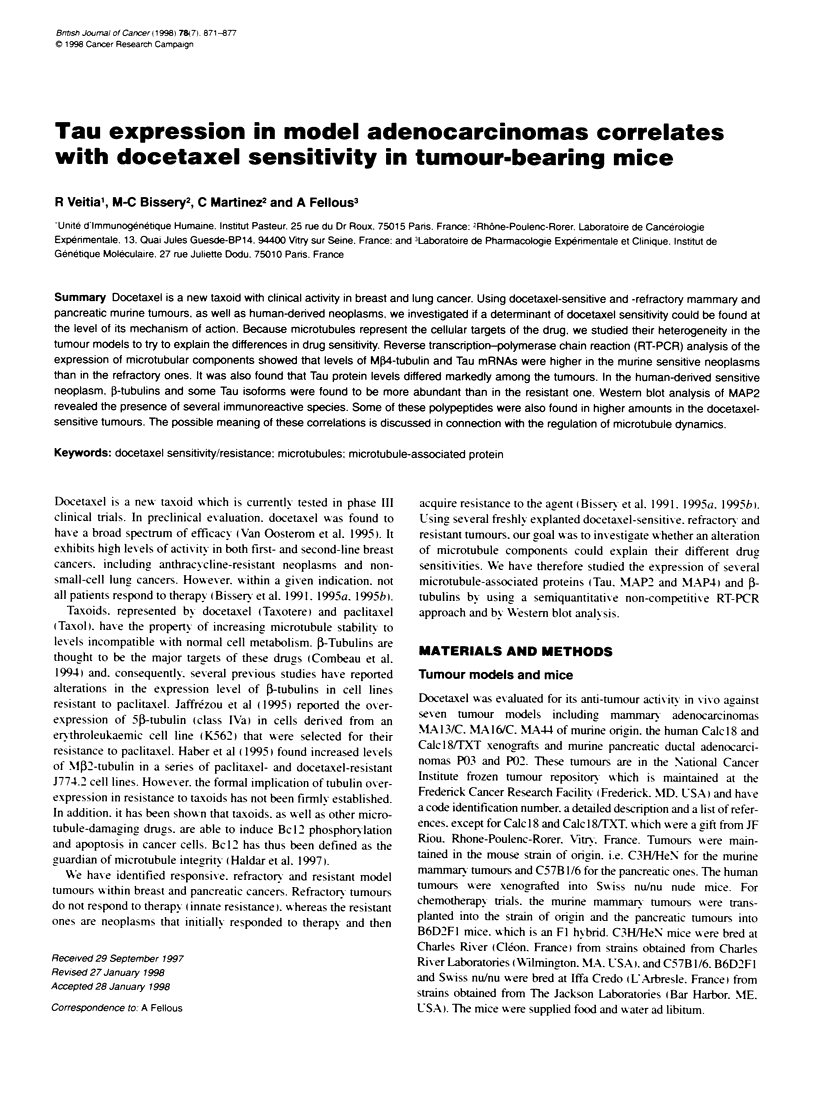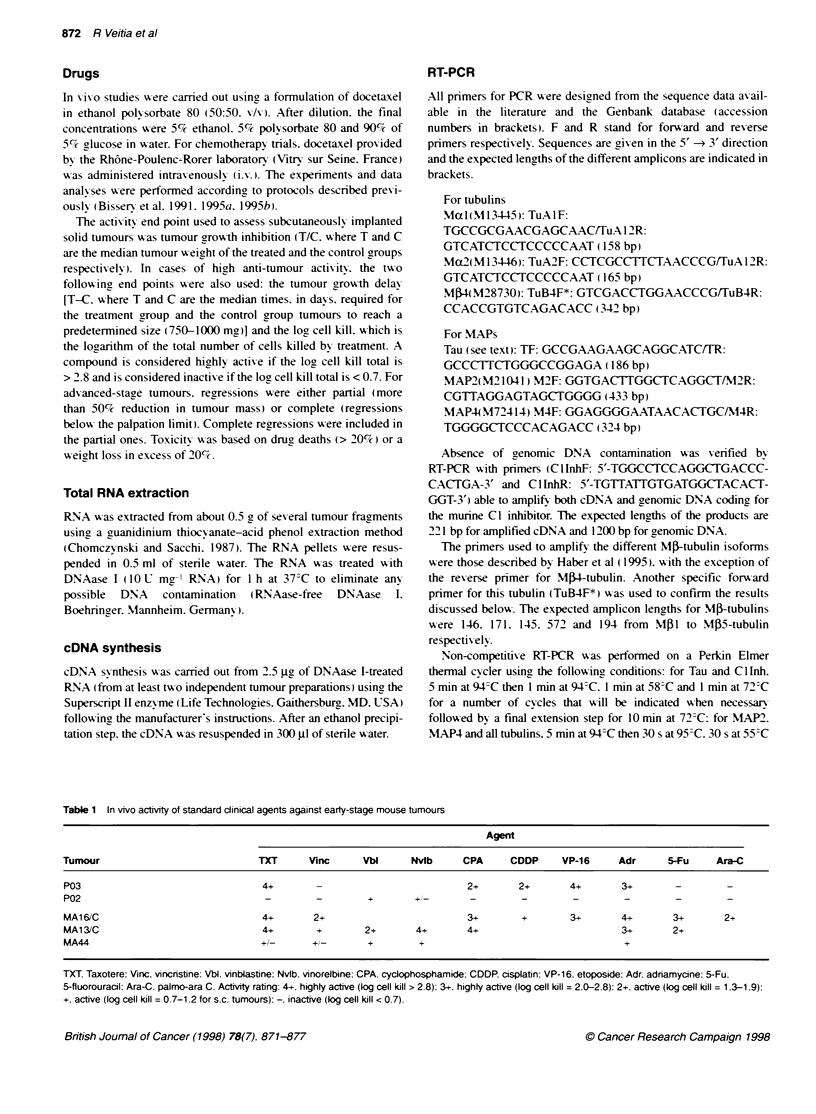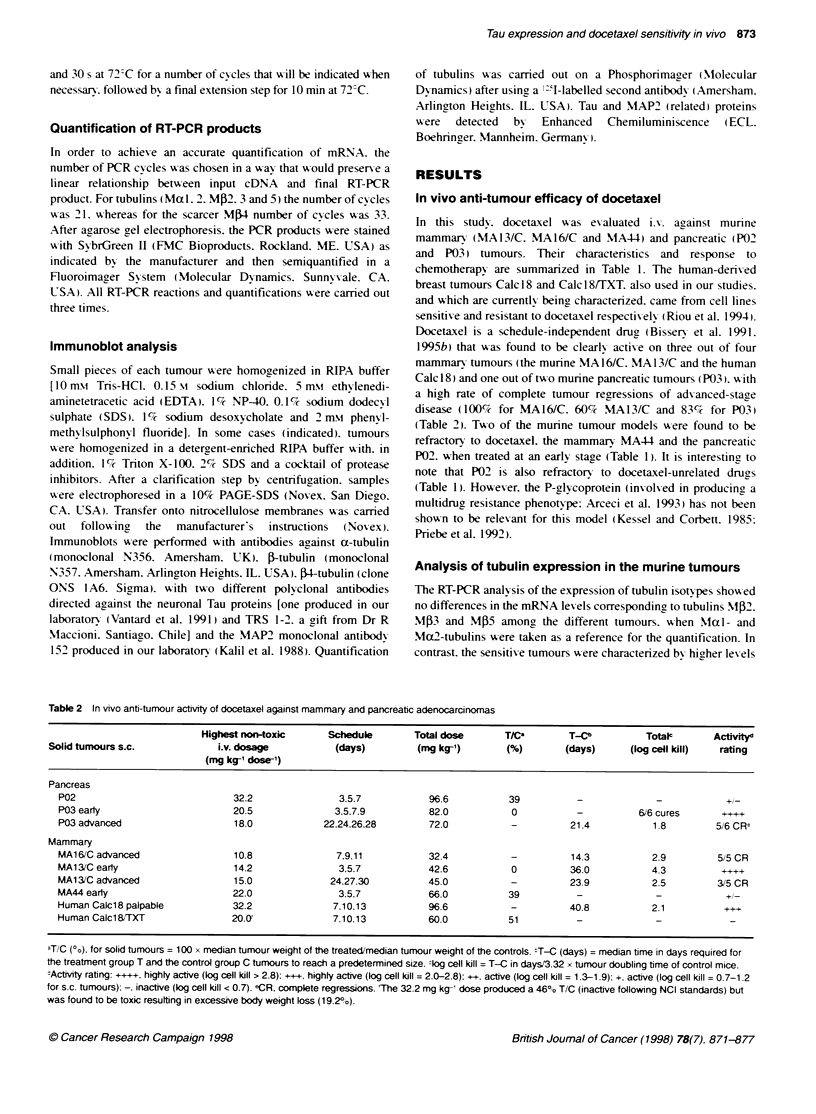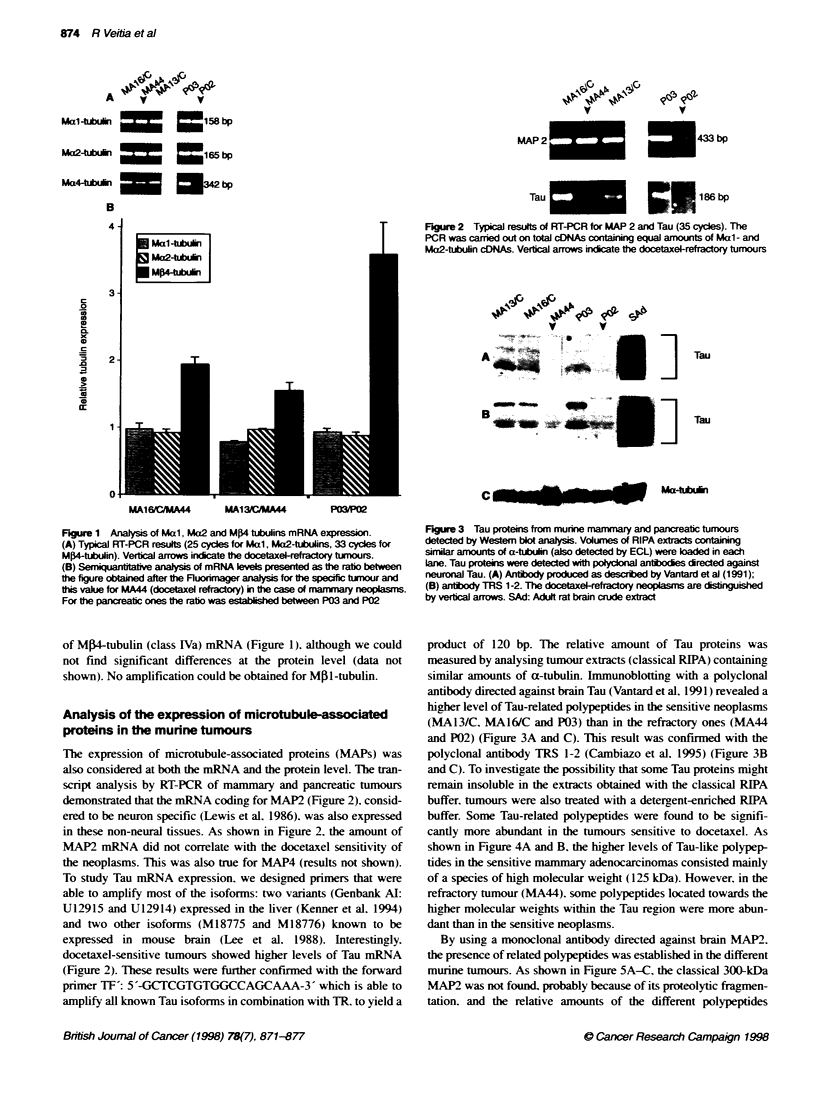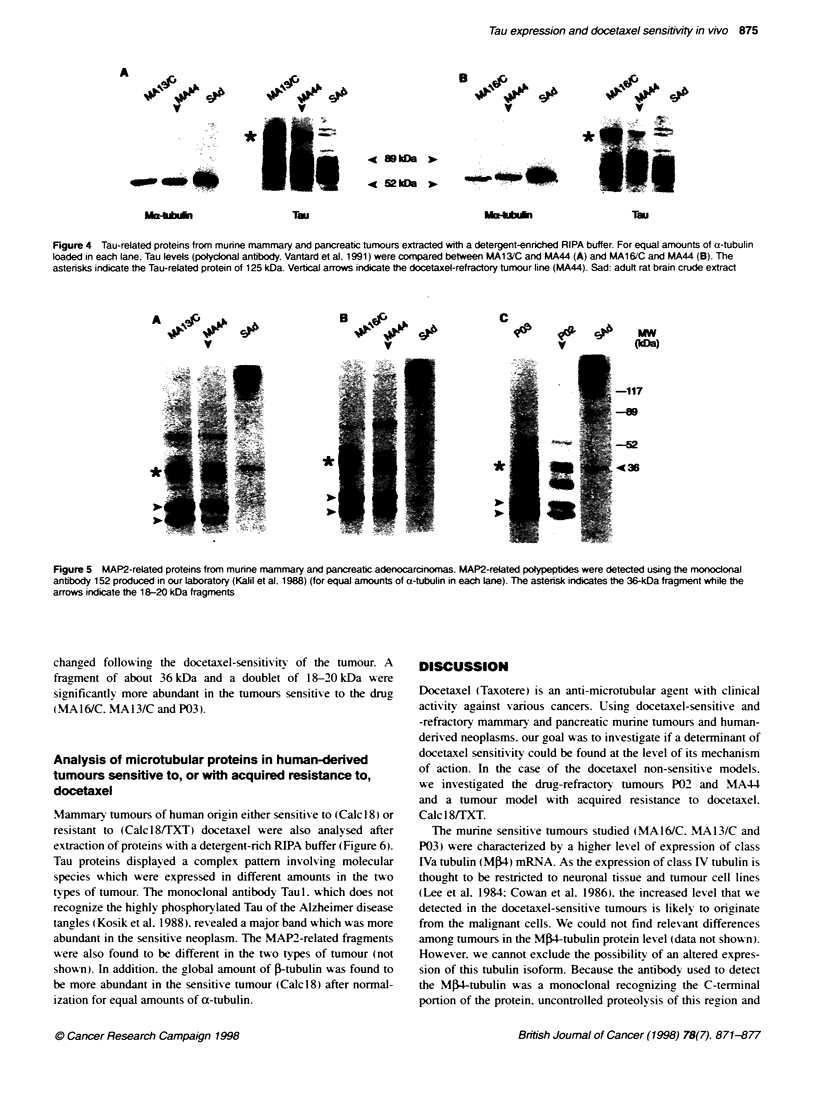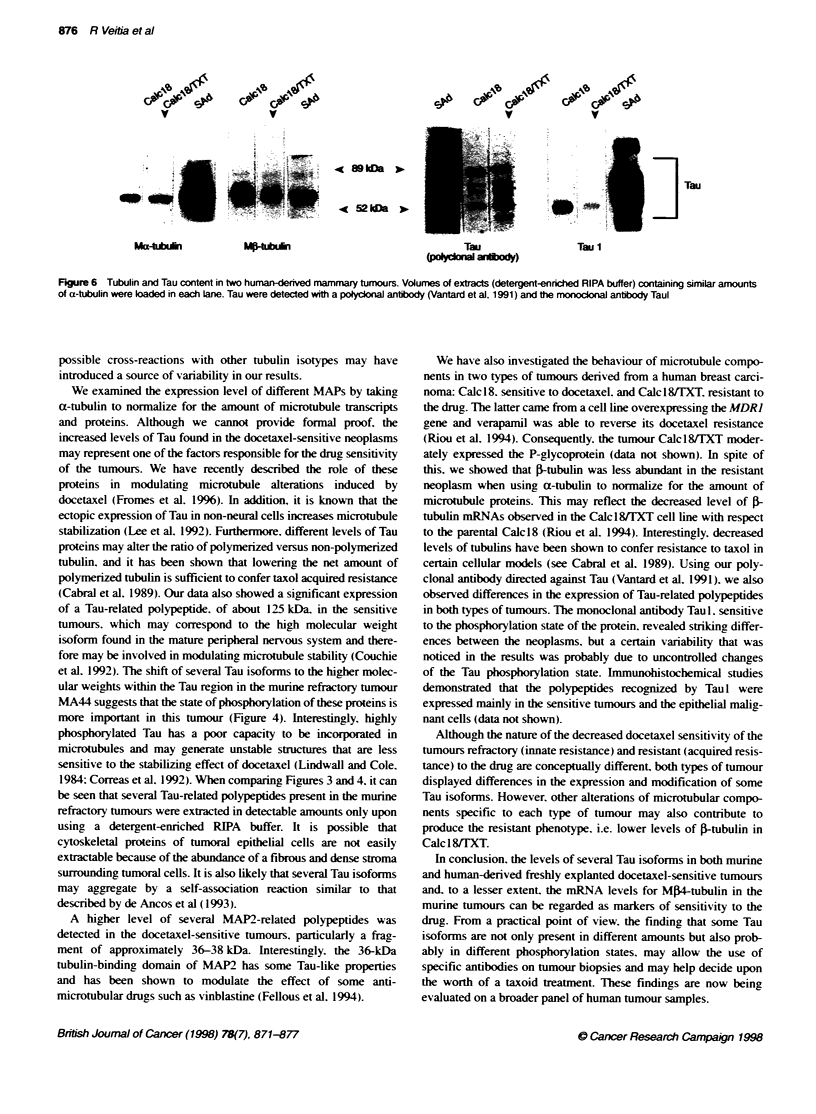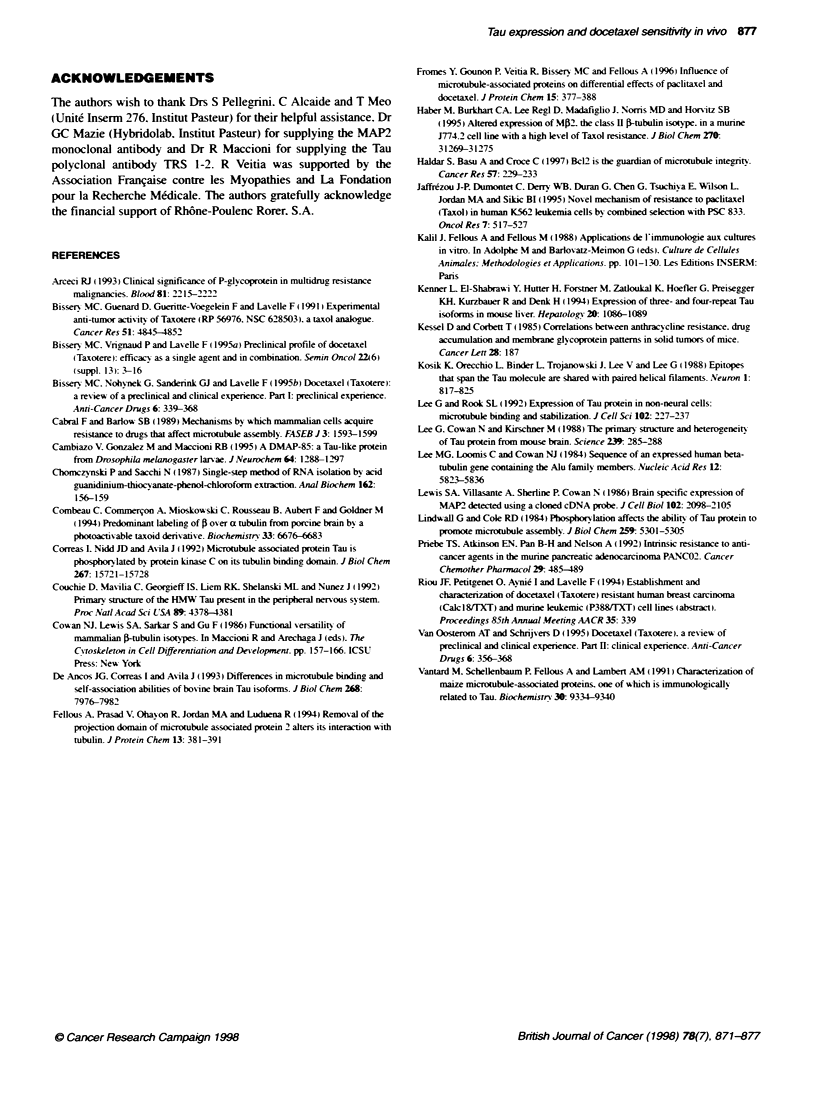# Tau expression in model adenocarcinomas correlates with docetaxel sensitivity in tumour-bearing mice.

**DOI:** 10.1038/bjc.1998.595

**Published:** 1998-10

**Authors:** R. Veitia, M. C. Bissery, C. Martinez, A. Fellous

**Affiliations:** Unité d'Immunogénétique Humaine, Institut Pasteur, Paris, France.

## Abstract

**Images:**


British Journal of Cancer (1998) 78(7), 871–877
© 1998 Cancer Research Campaign

### Tau expression in model adenocarcinomas correlates with docetaxel sensitivity in tumour-bearing mice

**R Veitia[1], M-C Bissery[2], C Martinez[2] and A Fellous[3]**

[1]Unité d'Immunogénétique Humaine, Institut Pasteur, 25 rue du Dr Roux, 75015 Paris, France; [2]Rhône-Poulenc-Rorer, Laboratoire de Cancérologie Expérimentale, 13, Quai Jules Guesde-BP14, 94400 Vitry sur Seine, France; and [3]Laboratoire de Pharmacologie Expérimentale et Clinique, Institut de Génétique Moléculaire, 27 rue Juliette Dodu, 75010 Paris, France

**Summary** Docetaxel is a new taxoid with clinical activity in breast and lung cancer. Using docetaxel-sensitive and -refractory mammary and pancreatic murine tumours, as well as human-derived neoplasms, we investigated if a determinant of docetaxel sensitivity could be found at the level of its mechanism of action. Because microtubules represent the cellular targets of the drug, we studied their heterogeneity in the tumour models to try to explain the differences in drug sensitivity. Reverse transcription–polymerase chain reaction (RT-PCR) analysis of the expression of microtubular components showed that levels of Mβ4-tubulin and Tau mRNAs were higher in the murine sensitive neoplasms than in the refractory ones. It was also found that Tau protein levels differed markedly among the tumours. In the human-derived sensitive neoplasm, β-tubulins and some Tau isoforms were found to be more abundant than in the resistant one. Western blot analysis of MAP2 revealed the presence of several immunoreactive species. Some of these polypeptides were also found in higher amounts in the docetaxel-sensitive tumours. The possible meaning of these correlations is discussed in connection with the regulation of microtubule dynamics.

**Keywords:** docetaxel sensitivity/resistance; microtubules; microtubule-associated protein

Docetaxel is a new taxoid which is currently tested in phase III clinical trials. In preclinical evaluation, docetaxel was found to have a broad spectrum of efficacy (Van Oosterom et al, 1995). It exhibits high levels of activity in both first- and second-line breast cancers, including anthracycline-resistant neoplasms and non-small-cell lung cancers. However, within a given indication, not all patients respond to therapy (Bissery et al, 1991, 1995*a*, 1995*b*).

Taxoids, represented by docetaxel (Taxotere) and paclitaxel (Taxol), have the property of increasing microtubule stability to levels incompatible with normal cell metabolism. β-Tubulins are thought to be the major targets of these drugs (Combeau et al, 1994) and, consequently, several previous studies have reported alterations in the expression level of β-tubulins in cell lines resistant to paclitaxel. Jaffrézou et al (1995) reported the over-expression of 5β-tubulin (class IVa) in cells derived from an erythroleukaemic cell line (K562) that were selected for their resistance to paclitaxel. Haber et al (1995) found increased levels of Mβ2-tubulin in a series of paclitaxel- and docetaxel-resistant J774.2 cell lines. However, the formal implication of tubulin over-expression in resistance to taxoids has not been firmly established. In addition, it has been shown that taxoids, as well as other microtubule-damaging drugs, are able to induce Bcl2 phosphorylation and apoptosis in cancer cells. Bcl2 has thus been defined as the guardian of microtubule integrity (Haldar et al, 1997).

We have identified responsive, refractory and resistant model tumours within breast and pancreatic cancers. Refractory tumours do not respond to therapy (innate resistance), whereas the resistant ones are neoplasms that initially responded to therapy and then acquire resistance to the agent (Bissery et al, 1991, 1995*a*, 1995*b*). Using several freshly explanted docetaxel-sensitive, refractory and resistant tumours, our goal was to investigate whether an alteration of microtubule components could explain their different drug sensitivities. We have therefore studied the expression of several microtubule-associated proteins (Tau, MAP2 and MAP4) and β-tubulins by using a semiquantitative non-competitive RT-PCR approach and by Western blot analysis.

*Received 29 September 1997*
*Revised 27 January 1998*
*Accepted 28 January 1998*

*Correspondence to:* A Fellous

### MATERIALS AND METHODS

### Tumour models and mice

Docetaxel was evaluated for its anti-tumour activity in vivo against seven tumour models including mammary adenocarcinomas MA13/C, MA16/C, MA44 of murine origin, the human Calc18 and Calc18/TXT xenografts and murine pancreatic ductal adenocarcinomas P03 and P02. These tumours are in the National Cancer Institute frozen tumour repository which is maintained at the Frederick Cancer Research Facility (Frederick, MD, USA) and have a code identification number, a detailed description and a list of references, except for Calc18 and Calc18/TXT, which were a gift from JF Riou, Rhone-Poulenc-Rorer, Vitry, France. Tumours were maintained in the mouse strain of origin, i.e. C3H/HeN for the murine mammary tumours and C57B1/6 for the pancreatic ones. The human tumours were xenografted into Swiss nu/nu nude mice. For chemotherapy trials, the murine mammary tumours were transplanted into the strain of origin and the pancreatic tumours into B6D2F1 mice, which is an F1 hybrid. C3H/HeN mice were bred at Charles River (Cléon, France) from strains obtained from Charles River Laboratories (Wilmington, MA, USA), and C57B1/6, B6D2F1 and Swiss nu/nu were bred at Iffa Credo (L'Arbresle, France) from strains obtained from The Jackson Laboratories (Bar Harbor, ME, USA). The mice were supplied food and water ad libitum.

### Drugs

In vivo studies were carried out using a formulation of docetaxel in ethanol polysorbate 80 (50:50, v/v). After dilution, the final concentrations were 5% ethanol, 5% polysorbate 80 and 90% of 5% glucose in water. For chemotherapy trials, docetaxel provided by the Rhône-Poulenc-Rorer laboratory (Vitry sur Seine, France) was administered intravenously (i.v.). The experiments and data analyses were performed according to protocols described previously (Bissery et al, 1991, 1995*a*, 1995*b*).

The activity end point used to assess subcutaneously implanted solid tumours was tumour growth inhibition (T/C, where T and C are the median tumour weight of the treated and the control groups respectively). In cases of high anti-tumour activity, the two following end points were also used: the tumour growth delay [T–C, where T and C are the median times, in days, required for the treatment group and the control group tumours to reach a predetermined size (750–1000 mg)] and the log cell kill, which is the logarithm of the total number of cells killed by treatment. A compound is considered highly active if the log cell kill total is > 2.8 and is considered inactive if the log cell kill total is < 0.7. For advanced-stage tumours, regressions were either partial (more than 50% reduction in tumour mass) or complete (regressions below the palpation limit). Complete regressions were included in the partial ones. Toxicity was based on drug deaths (> 20%) or a weight loss in excess of 20%.

### Total RNA extraction

RNA was extracted from about 0.5 g of several tumour fragments using a guanidinium thiocyanate–acid phenol extraction method (Chomczynski and Sacchi, 1987). The RNA pellets were resuspended in 0.5 ml of sterile water. The RNA was treated with DNAase I (10 U $mg^{-1}$ RNA) for 1 h at 37°C to eliminate any possible DNA contamination (RNAase-free DNAase I, Boehringer, Mannheim, Germany).

### cDNA synthesis cDNA synthesis was carried out from 2.5 μg of DNAase I-treated RNA (from at least two independent tumour preparations) using the Superscript II enzyme (Life Technologies, Gaithersburg, MD, USA) following the manufacturer's instructions. After an ethanol precipitation step, the cDNA was resuspended in 300 μl of sterile water.

### RT-PCR

All primers for PCR were designed from the sequence data available in the literature and the Genbank database (accession numbers in brackets). F and R stand for forward and reverse primers respectively. Sequences are given in the 5′ → 3′ direction and the expected lengths of the different amplicons are indicated in brackets.

For tubulins

Mα1(M13445): TuA1F: TGCCGCGAACGAGCAAC/TuA12R: GTCATCTCCTCCCCCAAT (158 bp)

Mα2(M13446): TuA2F: CCTCGCCTTCTAACCCG/TuA12R: GTCATCTCCTCCCCCAAT (165 bp)

Mβ4(M28730): TuB4F*: GTCGACCTGGAACCCG/TuB4R: CCACCGTGTCAGACACC (342 bp)

For MAPs
Tau (see text): TF: GCCGAAGAAGCAGGCATC/TR: GCCCTTCTGGGCCGGAGA (186 bp)
MAP2(M21041) M2F: GGTGACTTGGCTCAGGCT/M2R: CGTTAGGAGTAGCTGGGG (433 bp)
MAP4(M72414) M4F: GGAGGGGAATAACACTGC/M4R: TGGGGCTCCCACAGACC (324 bp)

Absence of genomic DNA contamination was verified by RT-PCR with primers (C1InhF: 5′-TGGCCTCCAGGCTGACCCCACTGA-3′ and C1InhR: 5′-TGTTATTGTGATGGCTACACTGGT-3′) able to amplify both cDNA and genomic DNA coding for the murine C1 inhibitor. The expected lengths of the products are 221 bp for amplified cDNA and 1200 bp for genomic DNA.

The primers used to amplify the different Mβ-tubulin isoforms were those described by Haber et al (1995), with the exception of the reverse primer for Mβ4-tubulin. Another specific forward primer for this tubulin (TuB4F*) was used to confirm the results discussed below. The expected amplicon lengths for Mβ-tubulins were 146, 171, 145, 572 and 194 from Mβ1 to Mβ5-tubulin respectively.

Non-competitive RT-PCR was performed on a Perkin Elmer thermal cycler using the following conditions: for Tau and C1Inh, 5 min at 94°C then 1 min at 94°C, 1 min at 58°C and 1 min at 72°C for a number of cycles that will be indicated when necessary followed by a final extension step for 10 min at 72°C; for MAP2, MAP4 and all tubulins, 5 min at 94°C then 30 s at 95°C, 30 s at 55°C

**Table 1** In vivo activity of standard clinical agents against early-stage mouse tumours

| | Agent | | | | | | | | | |
|---|---|---|---|---|---|---|---|---|---|---|
| **Tumour** | **TXT** | **Vinc** | **Vbl** | **Nvlb** | **CPA** | **CDDP** | **VP-16** | **Adr** | **5-Fu** | **Ara-C** |
| P03 | 4+ | – | | | 2+ | 2+ | 4+ | 3+ | – | – |
| P02 | – | – | + | +/– | – | – | – | – | – | – |
| MA16/C | 4+ | 2+ | | | 3+ | + | 3+ | 4+ | 3+ | 2+ |
| MA13/C | 4+ | + | 2+ | 4+ | 4+ | | | 3+ | 2+ | |
| MA44 | +/– | +/– | + | + | | | | + | | |

TXT, Taxotere; Vinc, vincristine; Vbl, vinblastine; Nvlb, vinorelbine; CPA, cyclophosphamide; CDDP, cisplatin; VP-16, etoposide; Adr, adriamycine; 5-Fu, 5-fluorouracil; Ara-C, palmo-ara C. Activity rating: 4+, highly active (log cell kill > 2.8); 3+, highly active (log cell kill = 2.0–2.8); 2+, active (log cell kill = 1.3–1.9); +, active (log cell kill = 0.7–1.2 for s.c. tumours); –, inactive (log cell kill < 0.7).

 © Cancer Research Campaign 1998

and 30 s at 72°C for a number of cycles that will be indicated when necessary, followed by a final extension step for 10 min at 72°C.

### Quantification of RT-PCR products

In order to achieve an accurate quantification of mRNA, the number of PCR cycles was chosen in a way that would preserve a linear relationship between input cDNA and final RT-PCR product. For tubulins (Mα1, 2, Mβ2, 3 and 5) the number of cycles was 21, whereas for the scarcer Mβ4 number of cycles was 33. After agarose gel electrophoresis, the PCR products were stained with SybrGreen II (FMC Bioproducts, Rockland, ME, USA) as indicated by the manufacturer and then semiquantified in a Fluoroimager System (Molecular Dynamics, Sunnyvale, CA, USA). All RT-PCR reactions and quantifications were carried out three times.

### Immunoblot analysis

Small pieces of each tumour were homogenized in RIPA buffer [10 mM Tris-HCl, 0.15 M sodium chloride, 5 mM ethylenediaminetetracetic acid (EDTA), 1% NP-40, 0.1% sodium dodecyl sulphate (SDS), 1% sodium desoxycholate and 2 mM phenylmethylsulphonyl fluoride]. In some cases (indicated), tumours were homogenized in a detergent-enriched RIPA buffer with, in addition, 1% Triton X-100, 2% SDS and a cocktail of protease inhibitors. After a clarification step by centrifugation, samples were electrophoresed in a 10% PAGE-SDS (Novex, San Diego, CA, USA). Transfer onto nitrocellulose membranes was carried out following the manufacturer's instructions (Novex). Immunoblots were performed with antibodies against α-tubulin (monoclonal N356, Amersham, UK), β-tubulin (monoclonal N357, Amersham, Arlington Heights, IL, USA), β4-tubulin (clone ONS 1A6, Sigma), with two different polyclonal antibodies directed against the neuronal Tau proteins [one produced in our laboratory (Vantard et al, 1991) and TRS 1-2, a gift from Dr R Maccioni, Santiago, Chile] and the MAP2 monoclonal antibody 152 produced in our laboratory (Kalil et al, 1988). Quantification of tubulins was carried out on a Phosphorimager (Molecular Dynamics) after using a $^{125}$I-labelled second antibody (Amersham, Arlington Heights, IL, USA). Tau and MAP2 (related) proteins were detected by Enhanced Chemiluminiscence (ECL, Boehringer, Mannheim, Germany).

### RESULTS

### In vivo anti-tumour efficacy of docetaxel

In this study, docetaxel was evaluated i.v. against murine mammary (MA13/C, MA16/C and MA44) and pancreatic (P02 and P03) tumours. Their characteristics and response to chemotherapy are summarized in Table 1. The human-derived breast tumours Calc18 and Calc18/TXT, also used in our studies, and which are currently being characterized, came from cell lines sensitive and resistant to docetaxel respectively (Riou et al, 1994). Docetaxel is a schedule-independent drug (Bissery et al, 1991, 1995*b*) that was found to be clearly active on three out of four mammary tumours (the murine MA16/C, MA13/C and the human Calc18) and one out of two murine pancreatic tumours (P03), with a high rate of complete tumour regressions of advanced-stage disease (100% for MA16/C, 60% MA13/C and 83% for P03) (Table 2). Two of the murine tumour models were found to be refractory to docetaxel, the mammary MA44 and the pancreatic P02, when treated at an early stage (Table 1). It is interesting to note that P02 is also refractory to docetaxel-unrelated drugs (Table 1). However, the P-glycoprotein (involved in producing a multidrug resistance phenotype: Arceci et al, 1993) has not been shown to be relevant for this model (Kessel and Corbett, 1985; Priebe et al, 1992).

### Analysis of tubulin expression in the murine tumours

The RT-PCR analysis of the expression of tubulin isotypes showed no differences in the mRNA levels corresponding to tubulins Mβ2, Mβ3 and Mβ5 among the different tumours, when Mα1- and Mα2-tubulins were taken as a reference for the quantification. In contrast, the sensitive tumours were characterized by higher levels

**Table 2** In vivo anti-tumour activity of docetaxel against mammary and pancreatic adenocarcinomas

| Solid tumours s.c. | Highest non-toxic i.v. dosage (mg kg$^{-1}$ dose$^{-1}$) | Schedule (days) | Total dose (mg kg$^{-1}$) | T/C[a] (%) | T–C[b] (days) | Total[c] (log cell kill) | Activity[d] rating |
|---|---|---|---|---|---|---|---|
| Pancreas | | | | | | | |
| P02 | 32.2 | 3.5.7 | 96.6 | 39 | – | – | +/– |
| P03 early | 20.5 | 3.5.7.9 | 82.0 | 0 | – | 6/6 cures | ++++ |
| P03 advanced | 18.0 | 22.24.26.28 | 72.0 | – | 21.4 | 1.8 | 5/6 CR[e] |
| Mammary | | | | | | | |
| MA16/C advanced | 10.8 | 7.9.11 | 32.4 | – | 14.3 | 2.9 | 5/5 CR |
| MA13/C early | 14.2 | 3.5.7 | 42.6 | 0 | 36.0 | 4.3 | ++++ |
| MA13/C advanced | 15.0 | 24.27.30 | 45.0 | – | 23.9 | 2.5 | 3/5 CR |
| MA44 early | 22.0 | 3.5.7 | 66.0 | 39 | – | – | +/– |
| Human Calc18 palpable | 32.2 | 7.10.13 | 96.6 | – | 40.8 | 2.1 | +++ |
| Human Calc18/TXT | 20.0[f] | 7.10.13 | 60.0 | 51 | – | – | – |

[a]T/C (%), for solid tumours = 100 × median tumour weight of the treated/median tumour weight of the controls. [b]T–C (days) = median time in days required for the treatment group T and the control group C tumours to reach a predetermined size. [c]log cell kill = T–C in days/3.32 × tumour doubling time of control mice. [d]Activity rating: ++++, highly active (log cell kill > 2.8); +++, highly active (log cell kill = 2.0–2.8); ++, active (log cell kill = 1.3–1.9); +, active (log cell kill = 0.7–1.2 for s.c. tumours); –, inactive (log cell kill < 0.7). [e]CR, complete regressions. [f]The 32.2 mg kg$^{-1}$ dose produced a 46% T/C (inactive following NCI standards) but was found to be toxic resulting in excessive body weight loss (19.2%).

© Cancer Research Campaign 1998 

**Figure 1** Analysis of Mα1, Mα2 and Mβ4 tubulins mRNA expression. (**A**) Typical RT-PCR results (25 cycles for Mα1, Mα2-tubulins, 33 cycles for Mβ4-tubulin). Vertical arrows indicate the docetaxel-refractory tumours. (**B**) Semiquantitative analysis of mRNA levels presented as the ratio between the figure obtained after the Fluorimager analysis for the specific tumour and this value for MA44 (docetaxel refractory) in the case of mammary neoplasms. For the pancreatic ones the ratio was established between P03 and P02

**Figure 2** Typical results of RT-PCR for MAP 2 and Tau (35 cycles). The PCR was carried out on total cDNAs containing equal amounts of Mα1- and Mα2-tubulin cDNAs. Vertical arrows indicate the docetaxel-refractory tumours

**Figure 3** Tau proteins from murine mammary and pancreatic tumours detected by Western blot analysis. Volumes of RIPA extracts containing similar amounts of α-tubulin (also detected by ECL) were loaded in each lane. Tau proteins were detected with polyclonal antibodies directed against neuronal Tau. (**A**) Antibody produced as described by Vantard et al (1991); (**B**) antibody TRS 1-2. The docetaxel-refractory neoplasms are distinguished by vertical arrows. SAd: Adult rat brain crude extract of Mβ4-tubulin (class IVa) mRNA (Figure 1), although we could not find significant differences at the protein level (data not shown). No amplification could be obtained for Mβ1-tubulin.

### Analysis of the expression of microtubule-associated proteins in the murine tumours

The expression of microtubule-associated proteins (MAPs) was also considered at both the mRNA and the protein level. The transcript analysis by RT-PCR of mammary and pancreatic tumours demonstrated that the mRNA coding for MAP2 (Figure 2), considered to be neuron specific (Lewis et al, 1986), was also expressed in these non-neural tissues. As shown in Figure 2, the amount of MAP2 mRNA did not correlate with the docetaxel sensitivity of the neoplasms. This was also true for MAP4 (results not shown). To study Tau mRNA expression, we designed primers that were able to amplify most of the isoforms: two variants (Genbank AI: U12915 and U12914) expressed in the liver (Kenner et al, 1994) and two other isoforms (M18775 and M18776) known to be expressed in mouse brain (Lee et al, 1988). Interestingly, docetaxel-sensitive tumours showed higher levels of Tau mRNA (Figure 2). These results were further confirmed with the forward primer TF′: 5′-GCTCGTGTGGCCAGCAAA-3′ which is able to amplify all known Tau isoforms in combination with TR, to yield a product of 120 bp. The relative amount of Tau proteins was measured by analysing tumour extracts (classical RIPA) containing similar amounts of α-tubulin. Immunoblotting with a polyclonal antibody directed against brain Tau (Vantard et al, 1991) revealed a higher level of Tau-related polypeptides in the sensitive neoplasms (MA13/C, MA16/C and P03) than in the refractory ones (MA44 and P02) (Figure 3A and C). This result was confirmed with the polyclonal antibody TRS 1-2 (Cambiazo et al, 1995) (Figure 3B and C). To investigate the possibility that some Tau proteins might remain insoluble in the extracts obtained with the classical RIPA buffer, tumours were also treated with a detergent-enriched RIPA buffer. Some Tau-related polypeptides were found to be significantly more abundant in the tumours sensitive to docetaxel. As shown in Figure 4A and B, the higher levels of Tau-like polypeptides in the sensitive mammary adenocarcinomas consisted mainly of a species of high molecular weight (125 kDa). However, in the refractory tumour (MA44), some polypeptides located towards the higher molecular weights within the Tau region were more abundant than in the sensitive neoplasms.

By using a monoclonal antibody directed against brain MAP2, the presence of related polypeptides was established in the different murine tumours. As shown in Figure 5A–C, the classical 300-kDa MAP2 was not found, probably because of its proteolytic fragmentation, and the relative amounts of the different polypeptides

© Cancer Research Campaign 1998

**Figure 4** Tau-related proteins from murine mammary and pancreatic tumours extracted with a detergent-enriched RIPA buffer. For equal amounts of α-tubulin loaded in each lane, Tau levels (polyclonal antibody. Vantard et al, 1991) were compared between MA13/C and MA44 (**A**) and MA16/C and MA44 (**B**). The asterisks indicate the Tau-related protein of 125 kDa. Vertical arrows indicate the docetaxel-refractory tumour line (MA44). Sad: adult rat brain crude extract

**Figure 5** MAP2-related proteins from murine mammary and pancreatic adenocarcinomas. MAP2-related polypeptides were detected using the monoclonal antibody 152 produced in our laboratory (Kalil et al, 1988) (for equal amounts of α-tubulin in each lane). The asterisk indicates the 36-kDa fragment while the arrows indicate the 18–20 kDa fragments changed following the docetaxel-sensitivity of the tumour. A fragment of about 36 kDa and a doublet of 18–20 kDa were significantly more abundant in the tumours sensitive to the drug (MA16/C, MA13/C and P03).

### Analysis of microtubular proteins in human-derived tumours sensitive to, or with acquired resistance to, docetaxel

Mammary tumours of human origin either sensitive to (Calc18) or resistant to (Calc18/TXT) docetaxel were also analysed after extraction of proteins with a detergent-rich RIPA buffer (Figure 6). Tau proteins displayed a complex pattern involving molecular species which were expressed in different amounts in the two types of tumour. The monoclonal antibody Tau1, which does not recognize the highly phosphorylated Tau of the Alzheimer disease tangles (Kosik et al, 1988), revealed a major band which was more abundant in the sensitive neoplasm. The MAP2-related fragments were also found to be different in the two types of tumour (not shown). In addition, the global amount of β-tubulin was found to be more abundant in the sensitive tumour (Calc18) after normalization for equal amounts of α-tubulin.

### DISCUSSION

Docetaxel (Taxotere) is an anti-microtubular agent with clinical activity against various cancers. Using docetaxel-sensitive and -refractory mammary and pancreatic murine tumours and human-derived neoplasms, our goal was to investigate if a determinant of docetaxel sensitivity could be found at the level of its mechanism of action. In the case of the docetaxel non-sensitive models, we investigated the drug-refractory tumours P02 and MA44 and a tumour model with acquired resistance to docetaxel, Calc18/TXT.

The murine sensitive tumours studied (MA16/C, MA13/C and P03) were characterized by a higher level of expression of class IVa tubulin (Mβ4) mRNA. As the expression of class IV tubulin is thought to be restricted to neuronal tissue and tumour cell lines (Lee et al, 1984; Cowan et al, 1986), the increased level that we detected in the docetaxel-sensitive tumours is likely to originate from the malignant cells. We could not find relevant differences among tumours in the Mβ4-tubulin protein level (data not shown). However, we cannot exclude the possibility of an altered expression of this tubulin isoform. Because the antibody used to detect the Mβ4-tubulin was a monoclonal recognizing the C-terminal portion of the protein, uncontrolled proteolysis of this region and

© Cancer Research Campaign 1998

**Figure 6** Tubulin and Tau content in two human-derived mammary tumours. Volumes of extracts (detergent-enriched RIPA buffer) containing similar amounts of α-tubulin were loaded in each lane. Tau were detected with a polyclonal antibody (Vantard et al, 1991) and the monoclonal antibody Taul possible cross-reactions with other tubulin isotypes may have introduced a source of variability in our results.

We examined the expression level of different MAPs by taking α-tubulin to normalize for the amount of microtubule transcripts and proteins. Although we cannot provide formal proof, the increased levels of Tau found in the docetaxel-sensitive neoplasms may represent one of the factors responsible for the drug sensitivity of the tumours. We have recently described the role of these proteins in modulating microtubule alterations induced by docetaxel (Fromes et al. 1996). In addition, it is known that the ectopic expression of Tau in non-neural cells increases microtubule stabilization (Lee et al. 1992). Furthermore, different levels of Tau proteins may alter the ratio of polymerized versus non-polymerized tubulin, and it has been shown that lowering the net amount of polymerized tubulin is sufficient to confer taxol acquired resistance (Cabral et al. 1989). Our data also showed a significant expression of a Tau-related polypeptide, of about 125 kDa, in the sensitive tumours, which may correspond to the high molecular weight isoform found in the mature peripheral nervous system and therefore may be involved in modulating microtubule stability (Couchie et al. 1992). The shift of several Tau isoforms to the higher molecular weights within the Tau region in the murine refractory tumour MA44 suggests that the state of phosphorylation of these proteins is more important in this tumour (Figure 4). Interestingly, highly phosphorylated Tau has a poor capacity to be incorporated in microtubules and may generate unstable structures that are less sensitive to the stabilizing effect of docetaxel (Lindwall and Cole, 1984; Correas et al. 1992). When comparing Figures 3 and 4, it can be seen that several Tau-related polypeptides present in the murine refractory tumours were extracted in detectable amounts only upon using a detergent-enriched RIPA buffer. It is possible that cytoskeletal proteins of tumoral epithelial cells are not easily extractable because of the abundance of a fibrous and dense stroma surrounding tumoral cells. It is also likely that several Tau isoforms may aggregate by a self-association reaction similar to that described by de Ancos et al (1993).

A higher level of several MAP2-related polypeptides was detected in the docetaxel-sensitive tumours, particularly a fragment of approximately 36–38 kDa. Interestingly, the 36-kDa tubulin-binding domain of MAP2 has some Tau-like properties and has been shown to modulate the effect of some anti-microtubular drugs such as vinblastine (Fellous et al. 1994).

We have also investigated the behaviour of microtubule components in two types of tumours derived from a human breast carcinoma: Calc18, sensitive to docetaxel, and Calc18/TXT, resistant to the drug. The latter came from a cell line overexpressing the *MDR1* gene and verapamil was able to reverse its docetaxel resistance (Riou et al. 1994). Consequently, the tumour Calc18/TXT moderately expressed the P-glycoprotein (data not shown). In spite of this, we showed that β-tubulin was less abundant in the resistant neoplasm when using α-tubulin to normalize for the amount of microtubule proteins. This may reflect the decreased level of β-tubulin mRNAs observed in the Calc18/TXT cell line with respect to the parental Calc18 (Riou et al. 1994). Interestingly, decreased levels of tubulins have been shown to confer resistance to taxol in certain cellular models (see Cabral et al. 1989). Using our polyclonal antibody directed against Tau (Vantard et al. 1991), we also observed differences in the expression of Tau-related polypeptides in both types of tumours. The monoclonal antibody Tau1, sensitive to the phosphorylation state of the protein, revealed striking differences between the neoplasms, but a certain variability that was noticed in the results was probably due to uncontrolled changes of the Tau phosphorylation state. Immunohistochemical studies demonstrated that the polypeptides recognized by Tau1 were expressed mainly in the sensitive tumours and the epithelial malignant cells (data not shown).

Although the nature of the decreased docetaxel sensitivity of the tumours refractory (innate resistance) and resistant (acquired resistance) to the drug are conceptually different, both types of tumour displayed differences in the expression and modification of some Tau isoforms. However, other alterations of microtubular components specific to each type of tumour may also contribute to produce the resistant phenotype, i.e. lower levels of β-tubulin in Calc18/TXT.

In conclusion, the levels of several Tau isoforms in both murine and human-derived freshly explanted docetaxel-sensitive tumours and, to a lesser extent, the mRNA levels for Mβ4-tubulin in the murine tumours can be regarded as markers of sensitivity to the drug. From a practical point of view, the finding that some Tau isoforms are not only present in different amounts but also probably in different phosphorylation states, may allow the use of specific antibodies on tumour biopsies and may help decide upon the worth of a taxoid treatment. These findings are now being evaluated on a broader panel of human tumour samples.

 © Cancer Research Campaign 1998

### ACKNOWLEDGEMENTS

The authors wish to thank Drs S Pellegrini, C Alcaide and T Meo (Unité Inserm 276, Institut Pasteur) for their helpful assistance, Dr GC Mazie (Hybridolab, Institut Pasteur) for supplying the MAP2 monoclonal antibody and Dr R Maccioni for supplying the Tau polyclonal antibody TRS 1-2. R Veitia was supported by the Association Française contre les Myopathies and La Fondation pour la Recherche Médicale. The authors gratefully acknowledge the financial support of Rhône-Poulenc Rorer, S.A.

### REFERENCES

Arceci RJ (1993) Clinical significance of P-glycoprotein in multidrug resistance malignancies. *Blood* **81**: 2215–2222

Bissery MC, Guenard D, Gueritte-Voegelein F and Lavelle F (1991) Experimental anti-tumor activity of Taxotere (RP 56976, NSC 628503), a taxol analogue. *Cancer Res* **51**: 4845–4852

Bissery MC, Vrignaud P and Lavelle F (1995*a*) Preclinical profile of docetaxel (Taxotere): efficacy as a single agent and in combination. *Semin Oncol* **22**(6) (suppl. 13): 3–16

Bissery MC, Nohynek G, Sanderink GJ and Lavelle F (1995*b*) Docetaxel (Taxotere): a review of a preclinical and clinical experience. Part I: preclinical experience. *Anti-Cancer Drugs* **6**: 339–368

Cabral F and Barlow SB (1989) Mechanisms by which mammalian cells acquire resistance to drugs that affect microtubule assembly. *FASEB J* **3**: 1593–1599

Cambiazo V, Gonzalez M and Maccioni RB (1995) A DMAP-85: a Tau-like protein from *Drosophila melanogaster* larvae. *J Neurochem* **64**: 1288–1297

Chomczynski P and Sacchi N (1987) Single-step method of RNA isolation by acid guanidinium-thiocyanate-phenol-chloroform extraction. *Anal Biochem* **162**: 156–159

Combeau C, Commerçon A, Mioskowski C, Rousseau B, Aubert F and Goldner M (1994) Predominant labeling of β over α tubulin from porcine brain by a photoactivable taxoid derivative. *Biochemistry* **33**: 6676–6683

Correas I, Nidd JD and Avila J (1992) Microtubule associated protein Tau is phosphorylated by protein kinase C on its tubulin binding domain. *J Biol Chem* **267**: 15721–15728

Couchie D, Mavilia C, Georgieff IS, Liem RK, Shelanski ML and Nunez J (1992) Primary structure of the HMW Tau present in the peripheral nervous system. *Proc Natl Acad Sci USA* **89**: 4378–4381

Cowan NJ, Lewis SA, Sarkar S and Gu F (1986) Functional versatility of mammalian β-tubulin isotypes. In Maccioni R and Arechaga J (eds). *The Cytoskeleton in Cell Differentiation and Development*. pp. 157–166. ICSU Press: New York

De Ancos JG, Correas I and Avila J (1993) Differences in microtubule binding and self-association abilities of bovine brain Tau isoforms. *J Biol Chem* **268**: 7976–7982

Fellous A, Prasad V, Ohayon R, Jordan MA and Luduena R (1994) Removal of the projection domain of microtubule associated protein 2 alters its interaction with tubulin. *J Protein Chem* **13**: 381–391

Fromes Y, Gounon P, Veitia R, Bissery MC and Fellous A (1996) Influence of microtubule-associated proteins on differential effects of paclitaxel and docetaxel. *J Protein Chem* **15**: 377–388

Haber M, Burkhart CA, Lee Regl D, Madafiglio J, Norris MD and Horvitz SB (1995) Altered expression of Mβ2, the class II β-tubulin isotype, in a murine J774.2 cell line with a high level of Taxol resistance. *J Biol Chem* **270**: 31269–31275

Haldar S, Basu A and Croce C (1997) Bcl2 is the guardian of microtubule integrity. *Cancer Res* **57**: 229–233

Jaffrézou J-P, Dumontet C, Derry WB, Duran G, Chen G, Tsuchiya E, Wilson L, Jordan MA and Sikic BI (1995) Novel mechanism of resistance to paclitaxel (Taxol) in human K562 leukemia cells by combined selection with PSC 833. *Oncol Res* **7**: 517–527

Kalil J, Fellous A and Fellous M (1988) Applications de l'immunologie aux cultures in vitro. In Adolphe M and Barlovatz-Meimon G (eds). *Culture de Cellules Animales: Methodologies et Applications*. pp. 101–130. Les Editions INSERM: Paris

Kenner L, El-Shabrawi Y, Hutter H, Forstner M, Zatloukal K, Hoefler G, Preisegger KH, Kurzbauer R and Denk H (1994) Expression of three- and four-repeat Tau isoforms in mouse liver. *Hepatology* **20**: 1086–1089

Kessel D and Corbett T (1985) Correlations between anthracycline resistance, drug accumulation and membrane glycoprotein patterns in solid tumors of mice. *Cancer Lett* **28**: 187

Kosik K, Orecchio L, Binder L, Trojanowski J, Lee V and Lee G (1988) Epitopes that span the Tau molecule are shared with paired helical filaments. *Neuron* **1**: 817–825

Lee G and Rook SL (1992) Expression of Tau protein in non-neural cells: microtubule binding and stabilization. *J Cell Sci* **102**: 227–237

Lee G, Cowan N and Kirschner M (1988) The primary structure and heterogeneity of Tau protein from mouse brain. *Science* **239**: 285–288

Lee MG, Loomis C and Cowan NJ (1984) Sequence of an expressed human beta-tubulin gene containing the Alu family members. *Nucleic Acid Res* **12**: 5823–5836

Lewis SA, Villasante A, Sherline P, Cowan N (1986) Brain specific expression of MAP2 detected using a cloned cDNA probe. *J Cell Biol* **102**: 2098–2105

Lindwall G and Cole RD (1984) Phosphorylation affects the ability of Tau protein to promote microtubule assembly. *J Biol Chem* **259**: 5301–5305

Priebe TS, Atkinson EN, Pan B-H and Nelson A (1992) Intrinsic resistance to anti-cancer agents in the murine pancreatic adenocarcinoma PANC02. *Cancer Chemother Pharmacol* **29**: 485–489

Riou JF, Petitgenet O, Aynié I and Lavelle F (1994) Establishment and characterization of docetaxel (Taxotere) resistant human breast carcinoma (Calc18/TXT) and murine leukemic (P388/TXT) cell lines (abstract). *Proceedings 85th Annual Meeting AACR* **35**: 339

Van Oosterom AT and Schrijvers D (1995) Docetaxel (Taxotere), a review of preclinical and clinical experience. Part II: clinical experience. *Anti-Cancer Drugs* **6**: 356–368

Vantard M, Schellenbaum P, Fellous A and Lambert AM (1991) Characterization of maize microtubule-associated proteins, one of which is immunologically related to Tau. *Biochemistry* **30**: 9334–9340

© Cancer Research Campaign 1998